# Finite Element Analysis of Silver Nanorods, Spheres, Ellipsoids and Core–Shell Structures for Hyperthermia Treatment of Cancer

**DOI:** 10.3390/ma15051786

**Published:** 2022-02-26

**Authors:** Muhammad Usama Daud, Ghulam Abbas, Muhammad Afzaal, Muhammad Yasin Naz, Nazma Goher Fatima, Abdul Ghuffar, Muhammad Irfan, Mater H. Mahnashi, Stanislaw Legutko, Jana Petrů, Jiří Kratochvíl, Usama Muhammad Niazi

**Affiliations:** 1 Department of Physics, Faisalabad Campus, Riphah International University, Faisalabad 44000, Pakistan; usamadaaud@gmail.com (M.U.D.); nazma.gohar@riphahfsd.edu.pk (N.G.F.); abdul.ghuffar@riphahfsd.edu.pk (A.G.); 2 Department of Physics, University of Agriculture, Faisalabad 38040, Pakistan; yasin306@uaf.edu.pk; 3 Electrical Engineering Department, College of Engineering, Najran University, Najran 61441, Saudi Arabia; miditta@nu.edu.sa; 4 Department of Pharmaceutical Chemistry, College of Pharmacy, Najran University, Najran 11001, Saudi Arabia; matermaha@gmail.com; 5 Faculty of Mechanical Engineering, Poznan University of Technology, 60-965 Poznan, Poland; stanislaw.legutko@put.poznan.pl; 6 Faculty of Mechanical Engineering, VSB—Technical University of Ostrava, Poruba, 708 00 Ostrava, Czech Republic; jana.petru@vsb.cz (J.P.); jiri.kratochvil@vsb.cz (J.K.); 7 Department of Mechanical Engineering Technology, National Skills University, Islamabad 44000, Pakistan; uknixi@gmail.com

**Keywords:** COMSOL Multiphysics, hyperthermia, surface coating, finite element analyses, silver nanostructures

## Abstract

The finite element analysis technique was used to investigate the suitability of silver nanorods, spheres, ellipsoids and core–shell structures for the hyperthermia treatment of cancer. The temperature of the silver nanostructures was raised from 42 to 46 °C, in order to kill the cancerous cells. The time taken by the nanostructures to attain this temperature, with external source heating, was also estimated. The heat transfer module in COMSOL Multiphysics was used for the finite element analysis of hyperthermia, based on silver nanostructures. The thermal response of different shapes of silver nanostructures was evaluated by placing them inside the spherical domain of the tumor tissue. The proposed geometries were heated at different time intervals. Optimization of the geometries was performed to achieve the best treatment temperature. It was observed that silver nanorods quickly attain the desired temperature, as compared to other shapes. The silver nanorods achieved the highest temperature of 44.3 °C among all the analyzed geometries. Moreover, the central volume, used to identify the thermal response, was the maximum for the silver nano-ellipsoids. Thermal equilibrium in the treatment region was attained after 0.5 μs of heating, which made these structures suitable for hyperthermia treatment.

## 1. Introduction

**Figure 1:**
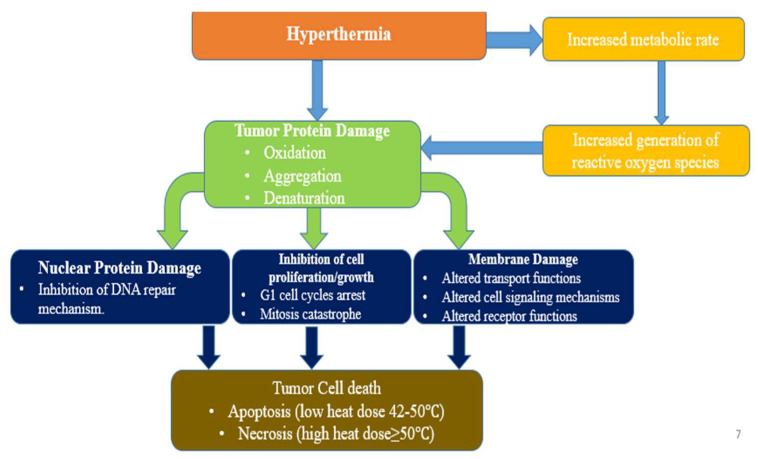
Mechanism of hyperthermia therapy of cancer cells.

Cancer is a multifactorial illness produced by a complex combination of hereditary and environmental variables [1,2,3]. Treating cancer is the most challenging health issue in the 21st century [4,5,6,7]. The cells of cancer are found to aggressively invade other bodily regions. These cells either form a tumor together, or can disseminate to the blood stream or lymph system [8,9]. These cells can metastasize to other organs and develop new tumors in places far away from the initial illness site. The ability of cancer cells to metastasize to other areas of the body is determined by a variety of factors, including blood flow and the type of cancer cells, and also the initial location of the cancer [8,9,10]. The substantial advancements in cancer research have resulted in a better understanding of cancer at the genetic, molecular, and cellular levels, allowing for novel therapeutic targets and procedures [11]. Cancer treatments include chemotherapy, surgery and radiation therapy [12,13]. Another way of treating cancer is by using hyperthermia, which involves heating the tumor region without damaging the normal cells [14]. Using hyperthermia, the temperature at the tumor location is raised to a certain level to kill the cancer cells [15]. The targeted tumor sites must attain a temperature in the range of 42–46 °C for hyperthermia to be therapeutically effective in cancer therapy [16]. Protein denaturation occurs when cells are exposed to this temperature range, resulting in a high fraction of co-aggregated denatured proteins [15]. Furthermore, a high temperature influences the cellular structure function and alters intracellular processes, ultimately leading to cancer mortality [16,17], as illustrated in Figure 1.

**Figure 2:**
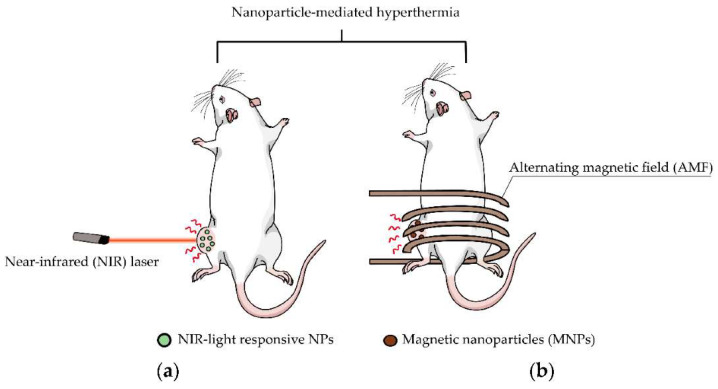
Schematic of hyperthermia utilizing nanoparticles: (**a**) photothermal treatment with laser irradiation to the cancer site, and (**b**) magnetic hyperthermia therapy with magnetic field exposure [21].

Figure 2 shows the following two examples of hyperthermia-mediated cancer treatment: photothermal therapy (PTT) and magnetic hyperthermia therapy (MHT) [18,19,20,21]. Insufficient blood flow, nourishment and oxygen supply are established inside the blood vessels in the tumor environment, due to the rapid temperature change in malignant cells; however, tumors are more resistant to temperature fluctuations [18,19]. Hyperthermia has been used in cancer therapy to improve the therapeutic effectiveness. 

Whole-body hyperthermia, local hyperthermia and regional hyperthermia are all possible through hyperthermia therapy [7]. Whole-body hyperthermia involves heating through an external heat source, such as microwaves or radio frequencies, which may have unhealthy side effects because of non-selective heating through non-selective process [21]. Regional hyperthermia heats a large area of cells, such as a body cavity, a limb or an organ. The regional perfusion technique, or the continuous hyperthermic peritoneal perfusion (CHPP) strategy, can be used to apply regional hyperthermia. Drawing blood from the patient’s body, heating it, and then pumping it back into the damaged organ constitutes the regional perfusion approach. The CHPP technique is used to treat peritoneal cancers, including primary peritoneal mesothelioma and stomach cancer [22]. If present in small areas, the cancer cells are killed by introducing heat carriers (Fe, Co, Ni, Ag, Au, etc.) into the body. This is referred to as local body hyperthermia, where nanoparticles are used as heat susceptors [23]. 

The nanoparticles (NPs) can be heated through high-intensity focused ultrasounds, magnetic hyperthermia, microwave/radio frequencies and plasmonic photo-thermal therapy. The heating method is selected by considering the given conditions. Since all hyperthermia treatment techniques have drawbacks, there is a need to develop new cancer treatment approaches that are more effective and less harmful to the healthy cells. When a magnetic field, in alternating mode, is applied to the NPs in magnetic hyperthermia, electromagnetic energy is transformed into heat. The heating of NPs is possible due to three mechanisms, namely, frictional heating in an anisotropic magnetic particle, eddy currents that have high electrical conductivity [24], and hysteresis [25]. The effects of eddy currents are generally negligible, due to the small size of NPs. The heating process depends upon the shape, nature, size and thermal characteristics of the tissue, and the frequency and magnitude of the magnetic field.

In designing NPs for practical use, the major issue is the biocompatibilization and functionalization of the surface coating materials, along with the selection of a suitable core. Noble metals, long-chain organic ligands, inorganic polymers and organic polymers are some examples of surface coatings. The importance of such coatings is that these are used to anchor the functional groups, for example, biomarkers, peptides and antibodies. These coatings are also used to prevent clustering of NPs, due to interactions between particles, which eventually provides stability to the colloidal solutions prepared with NPs. Furthermore, these coatings enhance the biocompatibility of NPs for preventing the leakage of toxic ions from the magnetic core into the biological system [26,27].

Due to their strong magnetic characteristics, outstanding biocompatibility and low cost, magnetic iron oxide nanoparticles are the most extensively employed magnetic nanomaterials. Iron oxide nanoparticles are being researched for their applications in a variety of fields, including biomedical, environmental science, sensing, electronic devices and energy storage [28,29]. Several researcher groups have reported the potential uses of iron oxide nanoparticles in hyperthermia. Table 1 provides a comparison of past studies on different nanostructured materials for the treatment of cancer.

Silver nanoparticles (AgNPs) stand out among the metallic nanomaterials because of their uses in health-care products, textiles, consumer products, medical devices and biosensing, due to their unique physical and chemical characteristics. AgNPs show good thermal, optical and electrical properties and activity against fungus, bacteria, and even viruses [36,37,38]. Recently, AgNPs have piqued the interest of researchers in nanomedicine, since multiple studies have shown that these NPs can generate antitumoral effects in in vitro and in vivo tumor models, potentially benefiting a variety of oncotherapy modalities and diagnostic tools [25,26,27,28]. In addition to antibacterial properties, AgNPs have unique cytotoxic effects against mammalian cells, making silver-based nanoparticles potentially useful in tumor treatment. The therapeutic efficacy of AgNPs is based on their distinct way of inducing cell death in mammalian cells. Despite the physical and chemical features, such as size, shape and heterogeneity of the capping material, their mechanism of action to promote cancer cell death is quite deterministic [39]. AgNPs are assembled in endosomes after being taken up by endocytosis-related processes, and the organelles are subsequently guided to undergo lysosomal fusion. The lysosomal acidic environment causes an increase in the release of silver ions from AgNPs, which then unbalances cellular homeostasis and leads to apoptotic cell death, depending on the biological aspect of the targeted cell [40].

Crystalline and/or amorphous core–shell structures have distinct properties, along with their medical applications. There is gradual deposition, according to the Stöber method, of small gold colloids onto the surface of cores [41]. The gold nanoparticles then grow in number and consolidate by forming an isolated island, to create an imperfect, uneven coating, which eventually forms a continuous complete shell that surrounds the core. Wu et al. [35] conducted a theoretical study on magnetite nanoparticles to analyze the temperature distribution in the tumor. They concluded that the constant temperature was obtained after 200 s, which is a very long time. The motivation behind our work was to achieve the desired temperature in a shorter time period, by using AgNPs. A comparative study on the heat generation, using a single silver nanoparticle, nanosphere, nanorod and nano-ellipsoid, was conducted in the reported work. The volume of these shapes is set as the following: $V_{sphere}\;≅\;V_{rod}\;≅\;V_{ellipsoid}\;i.e.,\;V_{sphere}=\frac{4}{3}\pi r^{3\;}=33510.32\;{\mathrm{nm}}^{3},\;V_{ellipsoid}=\frac{4}{3}\pi abc=33401.41{\;\mathrm{nm}}^{3},\;V_{rod}=\frac{4}{3}\pi r^{2}\left(r+h\right)=33324.96{\;\mathrm{nm}}^{3}$. The thermal effect of heat propagation in the tumor cell, as well as the spatial–temporal distributions of temperature during the treatment, are described in this model. Furthermore, the effect of different coating materials, such as gold (Au) and polymer (PEG), is analyzed. The thickness of the shell is discovered to be an essential component that influences the thermal response through the thermal characteristics of the materials utilized in the treatment system. Finally, varying quantities of NPs are attached to the core surface to simulate an incomplete coating surface. The heat transfer module in COMSOL Multiphysics is used to simulate the heating process of the nanostructures, using finite element simulations [42].

## 2. Methods

A 0.5 μm spherical domain of tissue enclosed the nanoparticle. COMSOL Multiphysics was used to identify the spatial and temporal distribution of temperature in this domain. The major goal of this research was to examine the thermal response of living tissue when the heating source (silver nanostructure) was of various forms, i.e., nanosphere, nanorod, nano-ellipsoid and complex core–shell structure.

Due to differences in the structure and function of tissues, balancing thermal energy in different biological tissues is a difficult process. Energy balancing is affected by the relative relevance of a heat transfer technique, the relevant time scale of the deposited energy, and changes in the boundary and initial conditions [43]. In order to simulate the fundamental features of the thermal state of the organism (or its components), as well as the impacts of the boundary and initial conditions, simplifying assumptions are frequently required. The application of the energy conservation law to a control volume is typically the first step in the design of such models.

(1) $$Q_{gain}=Q_{storage}+Q_{loss}+W$$

where $Q_{gain}$ is the heat gained by the tissue, $Q_{storage}$ is the heat stored in the tissue, $Q_{loss}$ is heat loss by conduction and W is work conducted by the tissue. Heat loss by conduction or heat exchange with flowing fluids, as well as the work conducted by the tissue, balance out the heat storage. Heat gain and heat energy storage due to heat generated by unit tissue segment *q* (*r*, *t*) can be represented as an integral over the control volume, as follows:

(2) $$Q_{gain}=\int^{\;}q\left(r,t\right)dV$$

(3) $$Q_{storage}=\int^{\;}\rho c\left[\frac{\partial }{\partial t}T\left(r,t\right)\right]dV$$

When no inertia is present, the heat conduction process is often represented using the Fourier law for bio-heat transfer problems.

(4) $$Q_{conduction}=-\frac{k_{t}}{\Delta L_{t}}A_{t}\left(T_{1}-T_{2}\right),\;T_{1}>T_{2}$$

where $k_{t}$ is the thermal conductivity of tissue, with dimensions [W/m*K]. It is based on the biological materials’ microscopic structures. The differential form of the conduction component of heat flux is as follows:

(5) $$q_{cond.}=-k_{t}\frac{\partial }{\partial x}T_{t}\left(x,t\right)$$

The convection term is expressed as follows:

(6) $$q_{conv.}=h\left(T_{w}-T_{fluid}\right)$$

where *h* is known as the coefficient of heat transfer, and it depends upon fluid velocity. The heat transfer due to fluid flow should contribute to blood perfusion and flow distribution in the biological tissue, due to the diversity of living structures. The flow is proportional to the difference in artery (*T_art_* or *T_a_*) and venous (*T_ven_*) blood temperature.

(7) $$q_{b}=\omega_{b}\rho_{b}c_{b}\left(T_{art}-T_{ven}\right)$$

It is possible that blood flows extremely slowly in capillary beds to achieve perfect thermal equilibrium with the tissues. Therefore, the heat flux related to perfusion (Equation (7)) can be approximated as follows:

(8) $$q_{b}=\omega_{b}\rho_{b}c_{b}\left(T_{art}-T_{t}\right)\;\;$$

Under these conditions, the transfer of energy by the stream of blood throughout the entire volume is as follows:

(9) $$Q_{b}=\int^{\;}\omega_{b}\rho_{b}c_{b}\left[T_{art}\left(r,t\right)-T_{t}\left(r,t\right)\right]dV$$

Even though it is commonly observed in model formulations, the result of Equation (9) is not unconditional; in larger arteries, it would be invalid, owing to intense blood mixing. By analyzing the various heat transmission modes, it is possible to create a thermal energy balance by ignoring the work conducted by the tissue across an arbitrary volume element, as follows:

(10) $$\int^{\;}\rho_{t}c_{t}\frac{\partial }{\partial t}T_{t}\left(r,t\right)dV=\int^{\;}-k_{t}\nabla T_{t}\left(r,t\right)dV+\int^{\;}\omega_{b}\rho_{b}c_{b}\left[T_{art}\left(r,t\right)-T_{t}\left(r,t\right)\right]dV+\int^{\;}Q_{m}\left(r,t\right)dV\;$$

We obtain, in a one-dimensional scenario with a homogenously distributed source of metabolic heat, $Q_{m}$.

(11) $$\rho_{t}c_{t}\frac{\partial }{\partial t}T_{t}\left(x,t\right)=k_{t}\frac{\partial^{2}}{\partial x^{2}}T_{t}\left(x,t\right)+\omega_{b}\rho_{b}c_{b}\left[T_{art}\left(r,t\right)-T_{t}\left(r,t\right)\right]+Q_{m}\;$$

This is the most common form of the heat transfer equation for living organisms, also known as the bio-heat transfer equation. Through scaling and dimensional analysis, we seek to examine its components and assumptions, which may alter depending on the conditions imposed by the modelled item, as well as the contribution of the transport mechanism involved. The temperature distribution in the tumor cell can be modeled using the Fourier heat equation, as follows:

(12) $$\rho C_{p}\frac{\partial T}{\partial t}+\rho C_{p}u.\nabla T+\nabla .q=Q+Q_{bio}$$

where,

(13) q=−k∇T

(14) $$Q_{bio}=\rho_{b}C_{p,b}w_{b}\left(T_{b}-T\right)+Q_{met}$$

which results in the following:

(15) $$\delta_{tz}\rho C_{p}\frac{\partial T}{\partial t}+\nabla .\left(-k\nabla T\right)=\rho_{b}C_{b}\omega_{b}\left(T_{b}-T\right)+Q_{met}+Q_{ext}$$

where $C_{p}$ is tissue-specific heat capacity at constant pressure, ρ is tissue density, k is cell thermal conductivity, $\rho_{b}$ is blood density, which is $1000\;\mathrm{kg}/m^{3}$, $C_{b}$ is specific heat of blood, which is 4180 J/(kg∗K), $\omega_{b}$ is blood perfusion rate of value 0.0064 1/s, $T_{b}$ is arterial blood temperature, which is approximately equal to core body temperature, i.e., 37 ℃ [44], *T* is the local temperature, and $Q=10^{16}\;\left(W/m^{3}\right)$ is heat dissipated by the nanoparticles in the volume of the cell [45], $Q_{met}=5790\;W/m^{3}$ for cancerous cells [46,47,48,49,50] and $Q_{ext}$ is the heat generated by loss powers. Initially, the temperature of the tissue was taken as normal for the human body ($T_{i}=37\;℃$). $T_{i}\left(r,0\right)=T_{0i},\;\frac{\partial T_{i}\left(r,0\right)}{\partial t}=0,\;\mathrm{while}\;i=1,\;2$.

**Figure 3:**
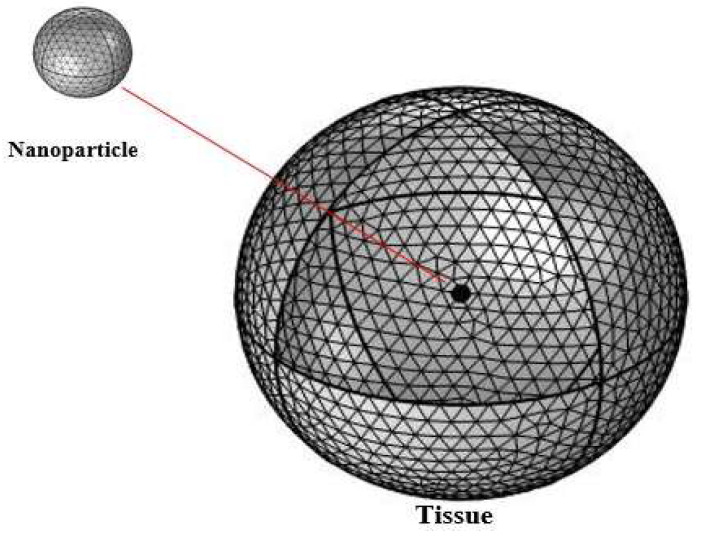
The discretized geometry of tissue and nanoparticle.

The geometry, which consisted of the tissue and nanoparticle, was discretized on all domains by free tetrahedral elements, as shown in Figure 3. There were 60,246 meshing domain elements after meshing the geometry, while the elemental size was chosen as finer. The following boundary conditions were applied to complete the procedure:

1. The tumor cell receives the heat flux from the particle in its entirety, i.e., continuity.
2. The temperature of the outer surface of the tissue is maintained at $T=T_{0}=37\;℃$.

**Figure 4:**
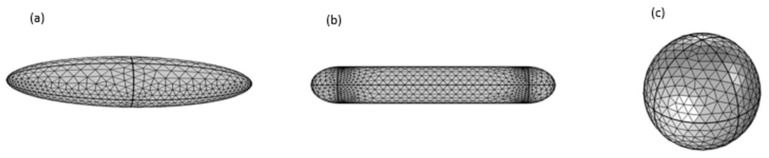
Different shapes used in simulations: (**a**) nano-ellipsoid, (**b**) nanorod and (**c**) nano-sphere.

**Figure 5:**
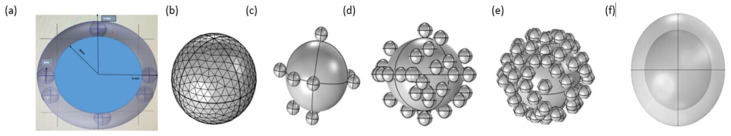
The core–shell structure simulated in COMSOL Multiphysics: (**a**) naked core with radius of 20 nm, with AgNPs with 4 nm radius attached to the core surface, (**b**) core (0 NP) and (**c**–**e**) 10, 40 and 70 nanoparticles attached to core, (**f**) complete shell (100 NPs).

Silver nanostructures of different shapes (sphere, rod and ellipsoid) were analyzed, as shown in Figure 4. Firstly, the spherical form of silver, with a radius of 20 nm, was analyzed. The volume of the particle was kept the same as the other shapes by computing the dimensions for the rod and ellipsoid. For the nanorod, the length of the cylinder was taken as $L_{cyl}=73\;\mathrm{nm}$, radius for the hemispherical caps $R_{cyl}=R_{cap}=11\;\mathrm{nm}$, and the dimensions for the ellipsoid were taken as 12−15−44.3 nm. To study the coating effect, the thickness of the Au and PEG polymer coating was set as 5, 10, 20, 30 and 40 nm. The core–shell structure, with a radius of 20 nm, was chosen for the purpose of analyzing the thermal evolution of the proposed structure. Three different coating surfaces (one spherical and two ellipsoidal) were also simulated on the silver nanoparticle core to compare the thermal responses of the ellipsoidal and spherical surface coatings. A silver nanoparticle, which provided a core with a radius of 20 nm, was coated with gold. The simulation growth of gold nanoparticles on the core of AgNP can be observed in Figure 5. The thermal properties of various materials used in this work are given in Table 2.

## 3. Results and Discussion

**Figure 6:**
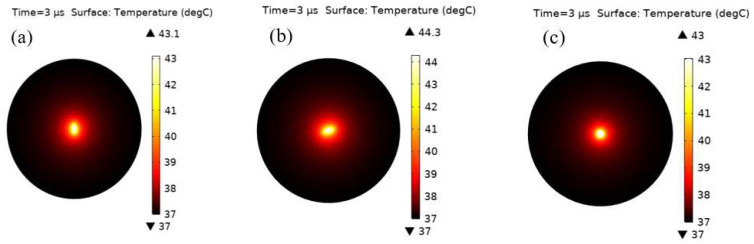
2D spatial temperature distributions in tumoral cell using (**a**) nano-ellipsoid, (**b**) nanorod and (**c**) nanosphere. The temperature distributions were obtained after 0.5 µm of heating.

**Figure 7:**
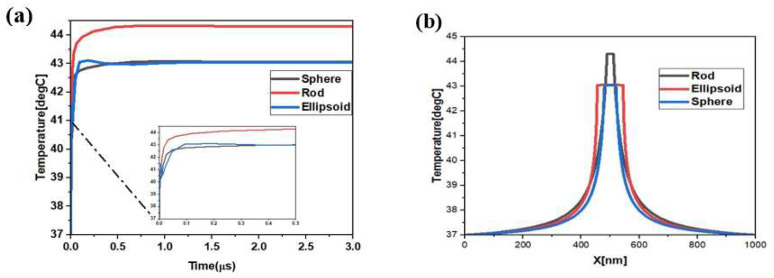
The time-dependent and radial–temporal distribution for three different shapes: (**a**) temperature evolution at the center of tumor (x = 0) and (**b**) the radial distribution after 3 µs of heating process.

To investigate the potential use of silver nanostructures, with different shapes, in hyperthermia, a nanorod, nano-ellipsoid and nanosphere were placed in a tumor with a spherical shape and a 500 nm radius. Their heating effect was simulated to kill the tumor cells. The first simulation showed the maximum temperature of all geometries in the tissue, when heated using an external source. The heating of the nanoparticles caused the temperature and thermal equilibrium of the tumoral cell to change over time, as observed in Figure 6. The temperatures attained by using various simulated forms differed notably. The variation in temperatures can be explained by the difference in the proportionate surface of each form, given that the thermal response is determined from the heat generation multiplied by the volume and surface ($Q=10^{8}\;\left(W/m^{2}\right)$. The maximum temperature, attained using the nanosphere, was 43 ℃. Similarly, the nano-ellipsoid and nanorod resulted in maximum attainable temperatures of 43.1 ℃ and 44.3 ℃, respectively. These temperature values were taken from the center of the particles, which then propagated into the surrounding medium, as shown in Figure 7. The thermal field distribution of an ellipse is larger than that of a sphere or a rod.

A functional surface is provided by the NPs coated with various materials in hyperthermia applications. The influence of the coating surface on thermal dissipation in the surrounding medium was investigated using simulations. The silver magnetic core was used as a heat source, with a polymer- or gold-like shell. The thermal conductivity coefficients for the two simulated shell materials resulted in opposing heat dissipation effects in relation to the shell thickness. The thermal conductivity for gold decreased, while it increased for the polymer.

**Figure 8:**
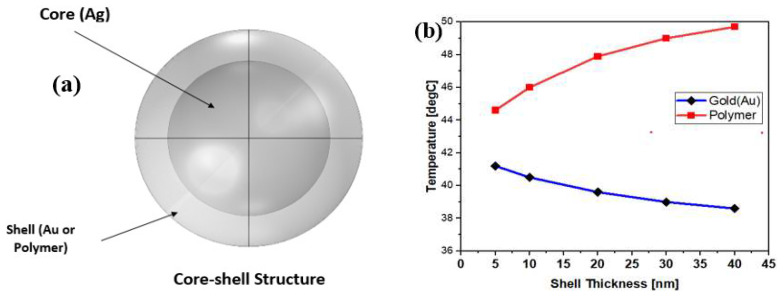
(**a**) The core–shell structure, (**b**) maximum temperature obtained by coating of gold and PEG polymer shell of thickness 5, 10, 20, 30 and 40 nm.

The hyperthermia process can be related to the functionality of core–shell structures. To achieve the maximum temperature, a sphere, with a radius of 20 nm, was chosen for the assigned nanoparticles. In this simulation, the core of AgNP was covered by the shell of gold or PEG polymer, and then the temperature induced by the core was analyzed. Different values of shell thickness were considered, and the effect of the temperature induced by the core of the nanoparticle is given in Figure 8. The temperature falls with an increase in the thickness of the Au shell, and rises with an increase in the thickness of the PEG polymer shell. As the thermal conductivity of gold is high, it rapidly transfers the heat to its surroundings. While the polymer, which has low thermal conductivity, preserves more heat inside the particle, which results in a rise in temperature. The effect and influence of the heat directly depend on the conductivity. These findings clearly show the possibility of controlling the temperature by changing the thickness of the shell. In addition, the material and shell thickness determine the required temperature for hyperthermia, which is dependent on the specific location of use in the human body.

**Figure 9:**
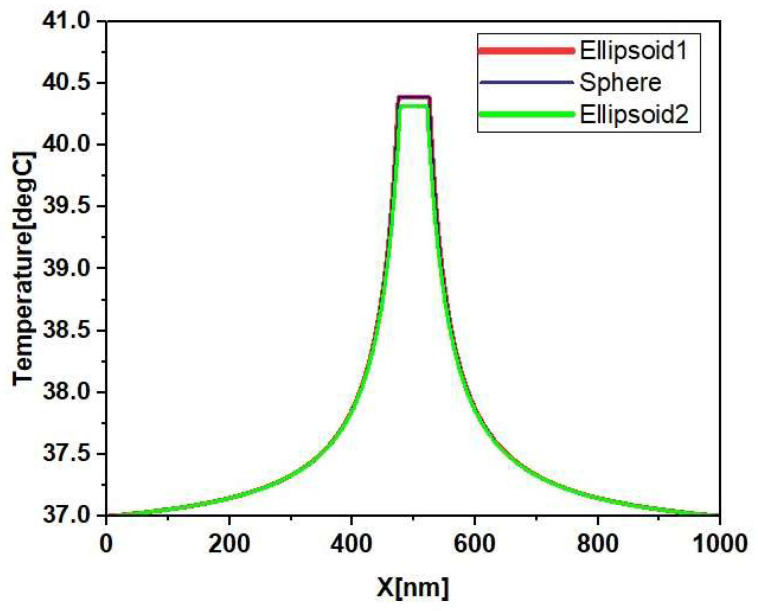
Radial temperature distributions of different coated shapes.

**Figure 10:**
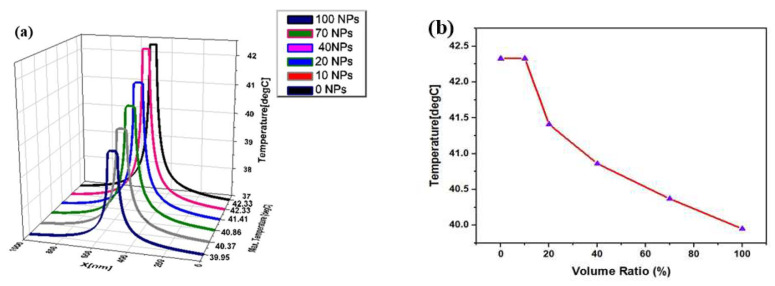
(**a**) Radial distribution of temperature after 3 µs of heating process for different levels of surface coating and (**b**) temperature evolution for different volume coverage ratios.

The shape of the coating surface plays an important role in the functionality of nanostructures. To study the effect of the coating surface, two forms of coating surfaces, namely, spherical (30 nm radius) and two ellipsoids ($ellipsoid_{1}:25-25-43.2\;\mathrm{nm},\;ellipsoid_{2}:22-25-49\;\mathrm{nm})$, were considered. From Figure 9, it is clear that there is no major difference in the temperature of the coating surfaces, which shows that anisotropy of coating surfaces is not important for the hyperthermia process, since gold nanoparticles grow and form an isolated island, to create an incomplete irregular coating, which is then transformed into a complete shell to cover the core. For this purpose, small AuNPs, with a 4 nm radius, were attached to the core surface of AgNPs, with a radius of 20 nm, which was then embedded into tissue with a 0.5 µm radius. The volume coverage ratio of AuNPs, compared to the volume of the full shell, was determined to describe the temperature profile for incompletely covered nanoparticles with varying amounts of nanoparticles attached, as shown in Figure 10. It is observed that the maximum temperature at the center of the nanoparticle was 42.3 ℃ for the naked core, while there was a gradual decrease in temperature with an increase in the amount of surface coating. The minimum temperature at the center of the nanoparticle was 39.9 ℃ when a complete shell was formed.

## 4. Conclusions

In this study, different nanostructures of silver were used for hyperthermia treatment, due to their non-toxic, antiviral and antibacterial properties. Different shapes, such as nanorod, nanosphere and nano-ellipsoid, were used in the simulations, and the simulations were performed for 3 µs. It was demonstrated that the temperature achieved by the silver nanorod was the maximum (i.e.,$\;T_{max}=44.3\;℃$), as compared to the other shapes. It was concluded that to observe the therapeutic benefits of nanoparticles, the size and shape are of key importance. The conducted research work can be utilized to optimize the thermal performance of silver nanoparticles for biomedical applications. A specific absorption rate of the thermal field for different shapes of nanoparticles, with the same volume, was studied. It was found that the maximum central volume was covered by the silver nano-ellipsoid, as compared to the other shapes, because of its thermal absorption rate. Thermal equilibrium was obtained after 0.5 µs from the beginning of the heating process. The effect of thickness and shape was analyzed, and it was observed that the thermal properties of the shell can be controlled by tailoring its thickness. The simulation analyzed the thermal response of the incomplete surface coating effect of different volume covering ratios of the gold shell. It was observed that the open surface that remains after the shell growth process is influenced by the temperature evolution. It was observed that silver nanorods quickly attain the desired temperature, as compared to other shapes. The silver nanorods gained the maximum temperature of 44.3 ℃ among all the analyzed geometries. Moreover, the central volume, used to identify the thermal response, was the maximum for the silver nano-ellipsoids. Thermal equilibrium in the treatment region was attained after 0.5 μs of heating, which made these structures suitable for hyperthermia treatment.

## Figures and Tables

**Table 1:** A comparison of the past studies utilizing nanostructured materials for the treatment of cancer.

Study Type	Particle Type and Size	Coating	Results and Drawbacks	Reference
Magnetic Hyperthermia (Experimental)	$3–4\;\mathrm{nm}\;\mathrm{of}\;{\mathrm{Fe}}_{2}O_{3}$	Dextran	Post-treatment tumor development is slowed. Induces inadvertent MF infiltration.	Jordan et al. (1997) [30]
Photo-thermal hyperthermia (theoretical)	Silica nano shell of 20 nm radius	Au coated	Suitable to obtain thermal regime. There is inconsideration of the role of blood perfusion rate and metabolic heat.	Dombrovsky et al. (2011) [31]
Theoretical model	${\mathrm{Fe}}_{3}O_{4}$ of 0.9 mm		The appropriate dose of nanoparticles for hyperthermia. The blood vessels close to the tumor reduce the temperature achieved in the tissue, whereas there are no large blood vessels around the tumor.	Pavel M. et al. (2009) [32]
Theoretical + experimental	${\mathrm{Fe}}_{3}O_{4}$ of 10 nm		The highest achievable temperature depends on surface-to-volume ratio. It is possible to describe patient-specific models.	Henrich F., Rahn H. and Odenbachs. (2015) [33]
Experimental Photothermal hyperthermia	Lipos AuNPs of 5–8 nm	Gold coated	Hybrid NPs (biodegradable) for treatment.	Rengan et al. (2015) [34]
Theoretical	${\mathrm{Fe}}_{3}O_{4}$ of 18 nm		They found that steady stable temperature achieved after 200 s was at the center of the tumor. The temperature curve declines with increasing distance from the centre at different exposure times.	Wu, L., Cheng, J., Liu, W., & Chen, X. (2015) [35]

**Table 2:** Thermal response of source materials used in this work.

	Thermal Conductivity [W/m*k]	Mass Density [kg/m^3^]	Specific Heat Capacity [J/kg*K]
Tissue [46]	0.512	1000	3800
Tumor [47]	71	21,500	132
Gold [48]	317	19,300	129
Silver [49]	429	10,500	235
Polymer [48]	0.2	1000	1000

## Data Availability

The reported data is available from the corresponding author on reasonable request.
